# Iron-Deficiency Anemia in an Elderly Caucasian Female: An Unusual Colonoscopic Finding

**DOI:** 10.7759/cureus.19564

**Published:** 2021-11-14

**Authors:** Roland Ngum, Angela Grigos, Magda Daoud

**Affiliations:** 1 Internal Medicine, Richmond University Medical Center, Staten Island, USA; 2 Internal Medicine/Gastroenterology, Richmond University Medical Center, Staten Island, USA

**Keywords:** helminthiasis, colonoscopy, elderly, anemia, iron-deficiency

## Abstract

Iron-deficiency anemia (IDA) is common in the elderly population. It is usually the result of chronic gastrointestinal diseases which could lead to iron losses, malabsorption, or both. IDA is most often the result of chronic gastrointestinal blood loss caused by esophagitis, gastritis, ulcer, colon cancer, pre-malignant polyps, or angiodysplasia. We are presenting a unique case that describes the unusual finding of intestinal helminthiasis in an elderly patient during endoscopic evaluation for IDA. It also touches on the risk factors, clinical manifestations, diagnosis, and treatment of enterobiasis*.*

## Introduction

The World Health Organization defines anemia as a hemoglobin level less than 13 g/dL in men and less than 12 g/dL in non-pregnant women [1]. It is a common presentation in the elderly population. In persons 65 years and older, anemia was present in 11.0% of men and 10.2% of women, with the prevalence rising to over 20% in people 85 years and older according to the third National Health and Nutrition Examination Survey (NHANES) [2]. Iron-deficiency anemia (IDA) in the elderly is usually the result of chronic gastrointestinal diseases which could lead to iron losses, malabsorption, or both. IDA is most often the result of chronic gastrointestinal blood loss caused by esophagitis, gastritis, ulcer, colon cancer or pre-malignant polyps, or angiodysplasia [3]. In one study, it correlates with low body mass index, low activity level, fair or poor self-reported health, frailty, congestive heart failure, and stroke or transient ischemic attack, and increased mortality risk [4]. We report a case of an 83-year-old Caucasian female who presented with severe IDA and had a colonoscopy done, which revealed intestinal helminthiasis.

## Case presentation

An 83-year-old female presented to the Emergency Room due to intermittent palpitations for the past one year but these got worse on the day of presentation. Palpitations were severe enough to wake her up from sleep. During the preceding three months, the patient had progressive fatigue and declining exercise intolerance. She could not walk more than a couple of blocks without having severe symptoms. The patient occasionally felt that she would “pass out” but never had any loss of consciousness. She denied any shortness of breath, chest pain, paroxysmal nocturnal dyspnea, blood loss including hematemesis, hematochezia, black stool or hematuria. The review of the system was also negative for anal pruritus, nausea, vomiting, change in bowel and bladder habits, cough or other respiratory symptoms, sleep disturbance, weight changes, or depressed mood. The patient stated that her appetite had been good and that she had been eating healthy.

Past medical history was significant for atrial fibrillation diagnosed one year ago. According to the patient, her last routine work-up was six months before presentation. The patient stated that there were no abnormal findings, but investigations and medical records were not available. Due to the prevailing COVID-19 pandemic, the patient missed one routine clinic visit. She had not had any prior endoscopic evaluation. Family history was negative for any inherited anemias, hemoglobinopathies, or bleeding disorders. The patient had no history of immunocompromise and was not on any steroids or other immunosuppressants. Her only medication was diltiazem extended-release 360 mg daily for atrial fibrillation. She was not on any anticoagulant or antiplatelet therapy. The patient was never exposed to any environment where she could have contracted a parasitic infestation. According to the patient, living conditions are clean, they drink bottled water at home and respect strict hygiene when handling food. There were no servants in the house. The family occasionally bought takeout food from nearby restaurants.

On examination, the temperature was 98.1°F, the heart rate 70 beats per minute, irregularly irregular, the blood pressure 125/68 mmHg sitting, 110/60 mmHg standing, the respiratory rate 16 breaths per minute, and the oxygen saturation 96% while the patient was breathing ambient air. The body-mass index was 28.7. The patient appeared pale, but in no acute distress and was able to speak in full sentences. The lungs were clear on auscultation. The first and second heart sounds (S1 and S2) were louder than normal and with a low pitched systolic murmur, 2/6 in intensity, heard in all four auscultatory areas. There were no gallops (S3 or S4). The heart rhythm was irregularly irregular. The peripheral pulses were bounding and irregularly irregular. The abdomen was nontender. A digital rectal examination revealed formed, brown stool but no blood. The remainder of the examination was normal.

On blood testing, the hemoglobin was 6.2 grams per deciliter (g/dL), which was confirmed on repeat testing. The hematocrit was 23.7%, mean corpuscular volume 65.8 femtoliters, and mean corpuscular hemoglobin 17.2 picograms. The reticulocyte count was 1.11%. Iron studies were done and showed serum iron of 12 micrograms per deciliter, the total iron-binding capacity of 491 micrograms per liter, ferritin of 4.4 nanograms per milliliter. A peripheral blood smear showed microcytosis, anisocytosis, and hypochromia. Blood type was A rhesus positive. Other test results are shown in Table 1. The fecal occult blood test was negative.

**Table 1:** Laboratory values of the patient

Test	Day 0	Day 3	Day 5	Reference values
	(admission)		(discharge)	
Hematology				
Red blood cell (million/µL)	3.60	4.22	3.96	3.9-5.2
Hemoglobin (g/dL)	6.2	8.3	8.0	11.2-15.7
Hematocrit (%)	23.7	29.9	28.3	34.0-45.0
Mean corpuscular volume (fl)	65.8	70.9	71.5	79-98
Mean corpuscular hemoglobin (pg)	17.2	19.6	20.2	25.0-32.0
Reticulocyte count (%)	1.11	-	-	0.9-2.5
Haptoglobin (mg/dL)	192	-	-	43-212
WBC (1,000/µL)	5.8	7.1	6.2	4.0-11.2
Eosinophils (absolute, 1,000/µL)	0.3	0.5	0.5	0.0-0.5
Eosinophils (%)	5.1	6.3	8.4	1-6
Platelets (1,000/µL)	385	357	310	150-400
Coagulation profile				
Prothrombin time (seconds)	14.2	-	-	12-14.8
International normalized ratio	1.15	-	-	0.9-1.12
Activated partial thromboplastin time (seconds)	28.4	-	-	22.8-36.5
Chemistry				
Iron (mcg/dL)	12	-	-	50-170
Total iron binding capacity(mcg/L)	491	-	-	250-450
% Saturation (%)	2.4	-	-	20-50
Ferritin (ng/dL)	4.4	-	-	10-291
Total bilirubin (mg/dL)	0.4	-	-	0.2-1.0
Folate(ng/mL)	10.3	-	-	1.1-20.0
Vitamin B12 (pg/dL)	398	-	-	211-911
Albumin (g/dL)	3.5	-	-	3.4-5.0
Blood urea nitrogen (mg/dL)	13	10	11	7-18
Creatinine (mg/dL)	0.8	0.8	0.7	0.55-1.02

**Figure 1:**
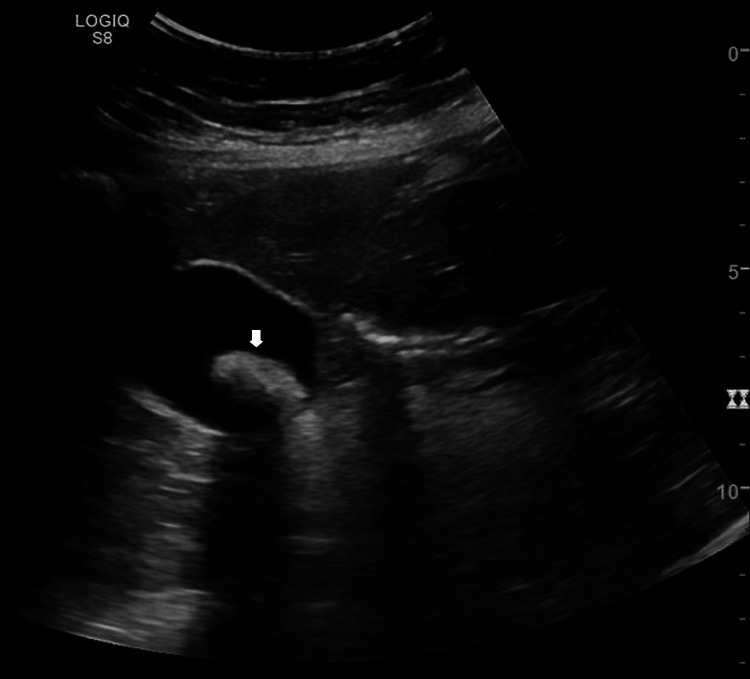
Abdominal ultrasound showing a gallstone (arrowhead)

Electrocardiography (ECG) showed atrial fibrillation with a ventricular rate of 88 beats per minute. There were no ST changes. A chest radiograph was normal. An ultrasound of the abdomen was positive for multiple gallstones with no signs of inflammation or bleeding (Figure 1).

The patient was transfused two units of packed red blood cells and the hemoglobin improved to 8.6 g/dL. She also received three doses of 100 mg of intravenous iron sucrose (100 mg of elemental iron). Oral therapy with 325 mg of ferrous sulfate (65 mg of elemental iron) was initiated. The patient was also given empiric pantoprazole 40 mg twice daily for eight weeks.

**Figure 2:**
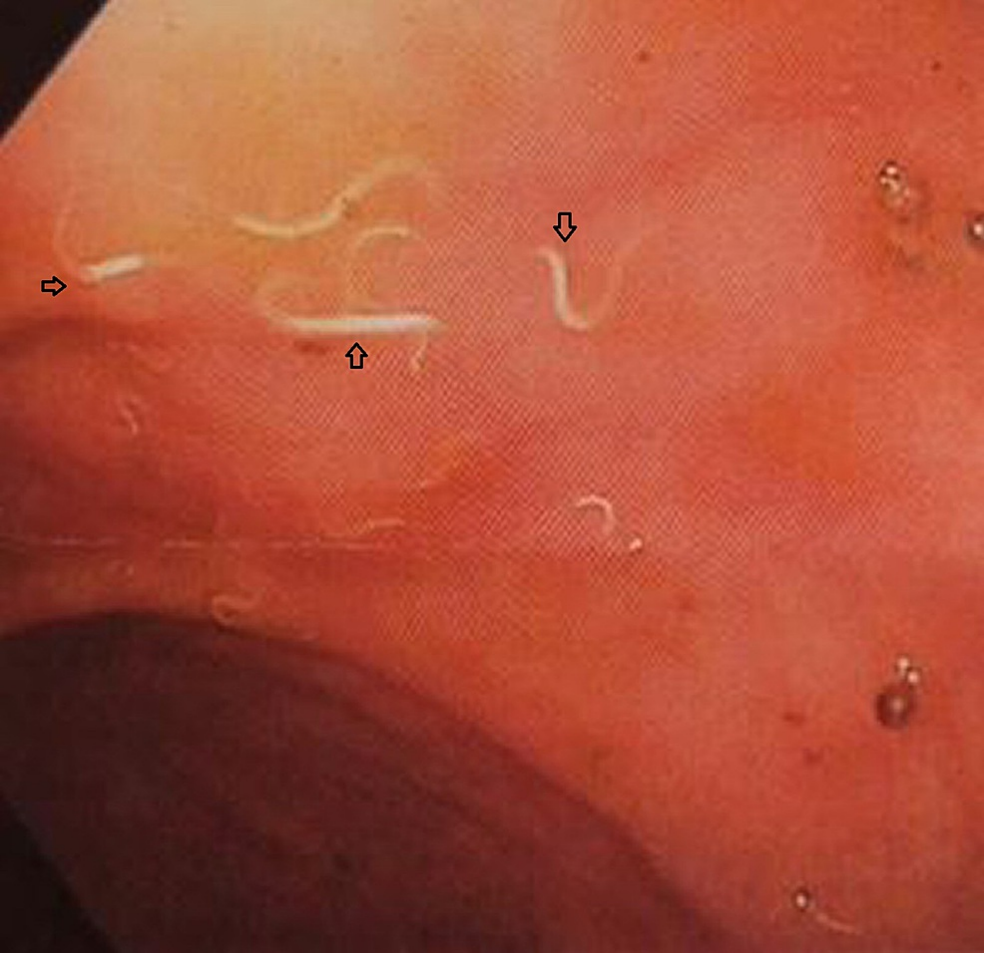
Colonoscopy showing helminths (arrowheads) in the sigmoid colon

**Figure 3:**
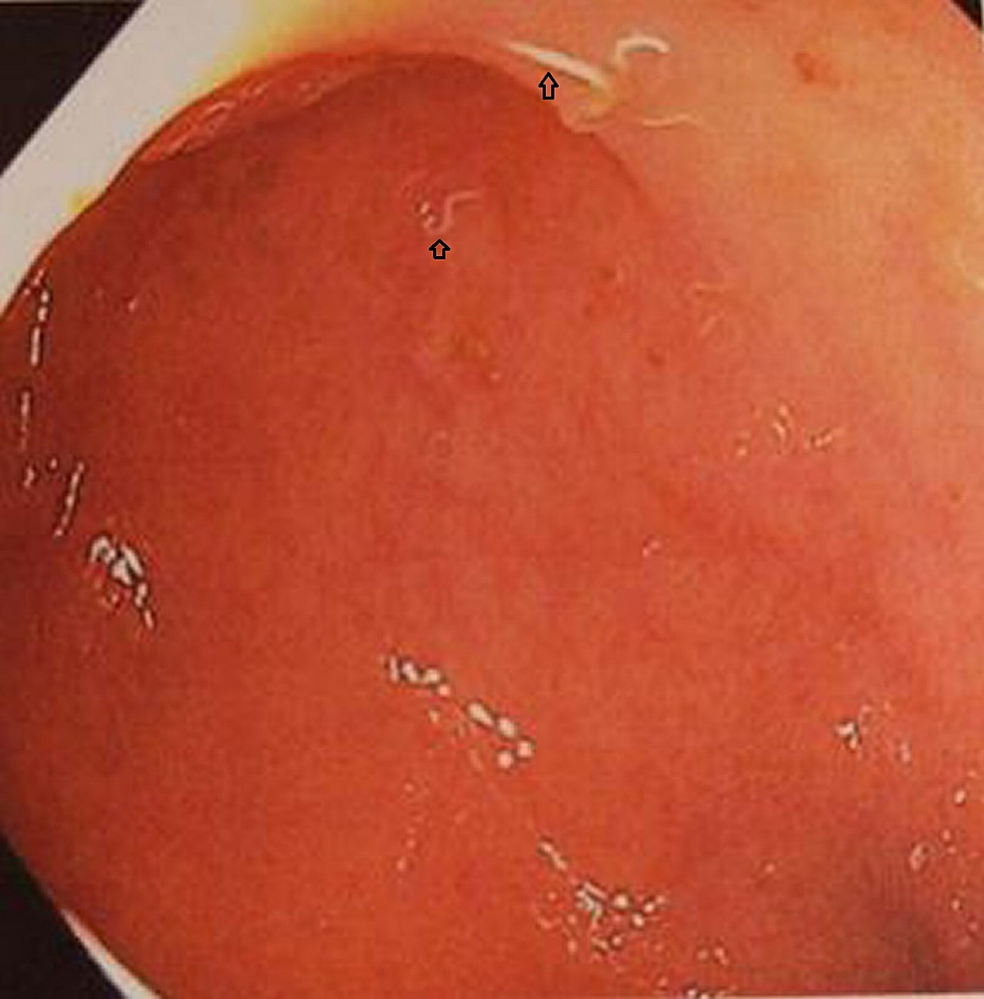
Colonoscopy showing helminths (arrowheads) in the rectum

Gastroenterology was consulted and the patient underwent an esophagogastroduodenoscopy (EGD) and colonoscopy on the third day of admission. The EGD showed medium-sized hiatal hernia and antral gastritis. Biopsies were taken which later showed chronic active gastritis, moderate in severity. A special stain (Giemsa) was positive for Helicobacter pylori microorganisms. Out of the blue, an excessive number of worms were found throughout the colon on colonoscopy (Figures 2, 3). These were estimated to measure 5-8mm in length. Other findings included small internal and external hemorrhoids, moderate diverticulosis in the sigmoid and descending colon. No bleeding or polyp was seen. Three stool aspirates were obtained and sent for microscopy which later came back negative. No biopsies were taken.

During the hospital stay, the patient’s symptoms of fatigue and palpitations improved. Upon discharge from the hospital, the patient was prescribed pyrantel pamoate 11 mg/kg single dose, to repeat in two weeks. Patient and family were advised to be treated simultaneously and to maintain strict hygienic conditions including washing of hands before and after handling food and after using the toilet. The patient was called after discharge and informed of the results of stool studies and biopsy. She agreed to follow up with the gastroenterologist for *Helicobacter pylori* eradication and with her primary care provider to confirm eradication of the pinworms (*Enterobius vermicularis)*. The patient was called a couple of months later and stated that she had not yet followed up with her primary care provider but that her condition had improved and she had no complaints. The entire household took the treatment simultaneously, the same dose.

## Discussion

This particular patient presented with fatigue, palpitations and was found to have severe anemia. Fatigue and palpitations are common presentations of severe anemia as seen in this patient. Tachycardia would be expected as a compensatory mechanism but this was absent in this patient. This could be due to blunted regulatory mechanisms that occur with aging. The same may hold true for orthostatic hypotension which was absent in this patient. Red blood cell indices indicated a microcytic and hypochromic anemia. This together with iron studies showing low ferritin are characteristic of IDA. A gastrointestinal cause of IDA was most likely in this patient. The prevalence of most gastrointestinal conditions associated with IDA increases with age, which is particularly true for neoplastic conditions [5] and angiodysplasia [6]. The next logical step was to rule out gastrointestinal blood loss. Endoscopy revealed multiple possible causes of chronic blood loss including gastritis, diverticulosis, and hemorrhoids. However, there was no active bleeding at the time of the procedure. Given the absence of gross bleeding or melena in the history, during hospitalization and on endoscopy, and a negative fecal occult blood test, these endoscopic findings were unlikely the cause of the anemia. Nonetheless, occult bleeding in the past could not be excluded.

The finding of numerous helminths in the colon was very unexpected in this patient residing in an urban environment in the United States. Groups at risk for intestinal helminthiasis include international students and travelers, migrant laborers, refugees, children of foreign adoptions, and the homeless [7]. Our patient did not fit into any of these categories. Eosinophilia would be expected but it was minimal to absent in this patient. Despite visual confirmation of intestinal helminths, the stool aspirate came back negative and no biopsies were taken. The specific helminth could not be identified. At the time the results were made available, the patient had been discharged as she has been waiting for already three days. During the waiting period, another stool sample could not be obtained as she did not have a bowel movement. A number of reasons could explain the false-negative stool test. Sample collection may have been inadequate. The patient had just had bowel prep for colonoscopy and stool aspirates would not be ideal for the diagnosis. In addition, if this patient had pinworms, a cellophane test would be the method of choice. This could not be done as a false negative stool test was not expected. Given the low prevalence of helminthiasis in developed countries, laboratory technicians may not be keen or well trained in their identification. Concentration techniques may boost the detection of eggs and identification of the specific helminthiasis. The differential diagnosis would include pinworms, hookworms, and *Ascaris*.

Pinworms are most likely in this patient given their epidemiology, size, location, and negative stool exam. Pinworms are the most common helminth infection in the United States (US) [7]. People affected in the US are commonly immigrants from developing countries or who have a compromised immune system [8]. Adult males and females measure 2-5 mm and 8-13 mm in length, respectively. The adults are located in the colon, mainly in the cecum and appendix [9]. Gravid females migrate to the perianal folds at night to lay eggs, hence the use of a cellophane test at night for improved yield. Most cases are asymptomatic but with increased worm burden, some cases may present with perianal itching, abdominal pain, nausea, and vomiting appendicitis, and eosinophilic enterocolitis. Though less common than hookworms, pinworms have been described in case reports as an incidental finding [10] and in association with IDA [11]. Colonoscopy can be useful for the diagnosis of parasitic infections, even if patients are asymptomatic [12]. 

Hookworms are the most commonly described helminths in association with IDA and this is mostly in tropical regions [13,14]. Hookworms are less common than pinworms in the US and adult worms are larger than pinworms and are located in the small intestine including the duodenum. EGD did not visualize any worms in this patient making the diagnosis less likely. However, they cannot be ruled out as the EGD cannot visualize the entire small intestine.

Currently approved medications for the treatment of enterobiasis include albendazole, mebendazole, and pyrantel pamoate. The choice of pyrantel pamoate was based on cost and accessibility. It is the cheapest of the three medications and it can be obtained over-the-counter. It would cost as low as $10 for a course of treatment per person and would be ideal for a large household. Two doses given two weeks apart were prescribed as recommended by the Centers for Disease Control and Prevention. Hygienic measures were reinforced to reduce the risk of re-infestation which is high in enterobiasis.

## Conclusions

In the endoscopic evaluation of IDA in an elderly patient, anything should be suspected and managed appropriately. Even when the diagnosis is compelling, biopsies should be taken to confirm it and to rule out other likely diagnoses. Incidental helminths, most likely pinworms were found in this patient and a cheap but effective over-the-counter treatment was started for her. Simultaneous treatment of her household was recommended to reduce disease transmission. Given the high rate of re-infestation, strict hygienic measures need to be implemented.
